# Cellular neurometabolism: a tentative to connect cell biology and metabolism in neurology

**DOI:** 10.1007/s10545-018-0226-8

**Published:** 2018-07-16

**Authors:** Àngels García-Cazorla, Jean-Marie Saudubray

**Affiliations:** 1 Neurometabolic Unit and Synaptic Metabolism Lab (Department of Neurology), Institut Pediàtric de Recerca. Hospital Sant Joan de Déu and CIBERER (ISCIII), Barcelona, Spain; 2 Department of Neurology, Neurometabolic Unit, Hopital Pitié Salpétrière, 47-83 Boulevard de l’Hopital, 75651 Paris Cedex 13, France

## Abstract

It has become increasingly evident that inborn errors of metabolism (IEMs) are particularly prevalent as diseases of the nervous system and that a broader, more inclusive definition of IEM is necessary. In fact, as long as biochemistry is involved, any kind of monogenic disease can become an IEM. This new, extended definition includes new categories and mechanisms, and as a general trend will go beyond a single biochemical pathway and/or organelle, and will appear as a connection of multiple crossroads in a system biology approach.

From one side, a simplified and updated classification of IEM is presented that mixes elements from the diagnostic approach with pathophysiological considerations into three large categories based on the size of molecules (“small and simple” or “large and complex”) and their implication in energy metabolism. But from another side, whatever their size, metabolites involved in IEM may behave in the brain as signalling molecules, structural components and fuels, and many metabolites have more than one role. Neurometabolism is becoming more relevant, not only in relation to these new categories of diseases but also as a necessary way to explain the mechanisms of brain damage in classically defined categories of IEM. Brain metabolism, which has been largely disregarded in the traditional approach to investigating and treating neurological diseases, is a major clue and probably the next imminent “revolution” in neurology and neuroscience. Biochemistry (metabolism) and cell neurobiology need to meet. Additionally, the brain should be studied as a system (connecting different levels of complexity).

**Electronic supplementary material:**

The online version of this article (10.1007/s10545-018-0226-8) contains supplementary material, which is available to authorized users.

## Introduction

Inborn errors of metabolism (IEMs) are particularly prevalent as diseases of the nervous system (Saudubray et al. 2016). Moreover, the whole group of neurometabolic disorders, many of them lacking biomarkers, is expected to experience substantial growth in the near future as a result of advanced genetic diagnostic techniques. In fact, as long as biochemistry is involved, any kind of monogenic disease can become an IEM. (Morava et al. 2015). These recently described diseases have challenged the traditional groups of IEMs and introduced new categories such as complex lipid biosynthesis and remodelling defects (Lamari et al. 2015) and congenital disorders of autophagy (Ebrahimi-Fakhari et al. 2016). The rapidly increasing amount of information available challenges our previous knowledge and will probably transform both neurology and metabolism by introducing new insights and therapeutic possibilities.

Descriptions of IEMs will go beyond a single biochemical pathway and/or organelle, and will appear as a connection of multiple crossroads in a system biology approach. Neurometabolism is becoming more relevant, not only in relation to these new categories of diseases but also as a necessary way to explain the mechanisms of brain damage in classically defined categories of IEM.

In order to complete this necessary transformation, we must change our approach to both biochemistry (metabolism) and cell neurobiology which means understanding the compartmentalised structure and interactions of neurons and non-neuronal cells, in addition to studying the brain as a system (connecting different levels of complexity).

## Part I—the evolving field of IEM and the increasing neurological involvement

### An extended definition of IEM as a concept

Metabolism involves thousands of proteins, mostly enzymes, receptors and transporters, the deficit of which causes IEM. Deficits can affect small or complex molecules. The number of IMD (inborn metabolic diseases) obviously depends on the definition of an IMD, and in the -omics era, this is changing quickly (Van Karnebeek et al. 2018). According to Morava, the “classification of a disorder as an IMD requires only that impairment of specific enzymes or biochemical pathways is intrinsic to the pathomechanism,” (Morava et al. 2015).

### Toward a new and simplified pathophysiological classification of IEM

Using this extended definition a recent tentative nosology of IEM encompasses more than 1100 IEM currently identified and provisionally classified into 130 groups (Ferreira et al. 2018). This list is of little help to clinicians and has little in common with neurologists’ clinical approach. A simplified and updated classification of IEM mixes elements from the practical diagnostic approach with pathophysiological considerations into three large categories based on the size of molecules (“small and simple” or “large and complex”) and their implication in energy metabolism (Saudubray and García-Cazorla 2018).

Whatever their size, metabolites involved in IEM may behave in the brain as signalling molecules, structural components and fuels, and many metabolites have more than one role (see supplemental material Fig. S1).

1. Disorders of small and simple molecules

Almost all these IEM have metabolic marker(s). Their diagnosis relies on plasma, urine, and CSF investigations. Many of them can be detected by neonatal metabolic screening. There are two subcategories in small molecule disorders defined by whether the phenotype primarily results from an accumulation or a deficiency.

1.1 Diseases linked to an accumulation: “Intoxication” disorders

Historically, the disorders in this group are the most typical IEM and are characterised by signs and symptoms resulting primarily from the abnormal accumulation of the compound(s) proximal to the block and potentially reverse as soon as the accumulation is removed. They share some characteristics:

- They do not interfere with embryo and foetal development and present after a symptom-free interval with clinical signs of intoxication (acute, intermittent, chronic and even progressive) provoked by intercurrent events and food intake.
- Most of these disorders are treatable.
- This group encompasses IEM of amino acid (AA) catabolism (PKU or MSUD), urea cycle defects, organic acidurias (MMA, GA1 etc.), carbohydrate intoxications metals accumulation and porphyrias (Saudubray and García-Cazorla 2018). Some purines/pyrimidines and metabolite repair defects (D/L-2-OH-glutaric, NADPH etc.) could be also included in this group.

In the brain, molecules that accumulate in intoxication disorders can behave as neurotransmitters (Kölker 2018) in the case of amino acids or stimulate biological pathways related to impaired autophagy and nerve growth factors. Synaptic plasticity and excitability are almost constantly impaired and executive functions are especially vulnerable. Therefore, and in spite of proper metabolic control, most of these patients display behavioural, emotional and learning difficulties.

1.2 Diseases linked to a deficiency

Symptoms result primarily from the defective synthesis of compounds distal to the block or from the defective transport of an essential molecule through intestinal epithelium, blood-brain barrier (Table 1 and Fig. S2 in supplemental material), and cytoplasmic or organelle membranes. Unlike those defects belonging to the intoxication group most of these defects interfere with embryofoetal development causing a neurodevelopmental disruption, have a congenital presentation and share many characteristics with disorders in the complex molecules group (see later). Molecular mechanisms of IEM linked to essential compounds are different from those linked to non-essential ones.

**Table 1:** Diseases of transport across the blood-brain-barrier. Mechanisms and symptoms

	Transport mechanism	Diseases	Symptoms
Glucose	Facilitated diffusion	GLUT-1 defect GLUT-10 (not glucose transport but a similar substance)	Epilepsy, ID, abnormal movements Arterial tortuosity syndrome, strokes
Lactate, ketone bodies	Diffusional, saturable cotransport with protons	MCT-1 defect	Episodes of severe ketoacidosis in early childhood
Amino acids	Large neutral aa transporter (L-system) Na+ dependent aa transport	BCAA defect (gene SLC7a5) Serine transport defect (gene SLC6a14)	ID, autism, epilepsy, microcephaly, develop delay, hypomyelination
Lipids		DHA transporter defect (gene Mfsd2a)	Microcephaly, brain malformation, early death

Abbreviations: *BCAA,* branched chain amino acids; *DHA*, docohexanoic acid; *ID*, intellectual disability

Essential compounds come from diet and must be transported through cellular membranes. Accordingly, IEM are linked to carrier defects (essential amino acids like *SLC 7A5* for BCAA (Tărlungeanu et al. 2016), or essential fatty acids (FA) such as *MFSD2A* for DHA (Guemez-Gamboa et al. 2015). BCDH kinase deficiency overactivates, causing irreversible BCAA oxidation in very low levels of BCAA as in *SLC 7A5* mutations and presents a similarly devastating neurological syndrome with neurodevelopmental disruption (Novarino et al. 2013).

Non-essential compounds can be synthesised inside cells. Their availability depends on the integrity of the synthesis pathway. IEM are linked to enzyme defects (such as serine, glutamine, and asparagine synthetase deficiency) (Häberle et al. 2005; Acuna-Hidalgo et al. 2015; Ruzzo et al. 2013). In addition to AA and FA synthesis and transport defects, this group also encompasses the IEM causing metal deficiency (Boycott et al. 2015; Park et al. 2015) as well as neurotransmitter metabolism and transport defects (Tristán-Noguero and García-Cazorla 2018, this issue). Some vitamin-related disorders and purine and pyrimidine defects also belong to this category.

Major neurodevelopmental disruptions lead to severe global encephalopathies where almost all neurological functions are chronically altered. In early onset presentations, patients display severe psychomotor delays affecting both motor and cognitive milestones. Microcephaly and hypomyelination are very common as epilepsy and movement disorders. These defects mimic early “non-metabolic” genetic encephalopathies that affect crucial neurodevelopmental functions such as neuronal precursor proliferation, migration, pruning and dendrite development. This is because these small molecules contribute to antenatal brain “construction” in terms of signalling, cytoskeleton guidance, synapse formation and later on in experience-dependent synapse remodelling.

2) Energy-related defects

These consist of IEM with symptoms due, at least in part, to a deficiency in energy production or utilisation within the liver, myocardium, muscle, brain and other tissues.

2.1 *Membrane carriers* of energetic molecules (glucose: GLUT, FA, ketone bodies, monocarboxylic acids: MCT) display many tissue specific isozymes as GLUT-1 and MCT-1.
2.2 *Mitochondrial defects* encompass aerobic glucose oxidation defects presenting with congenital lactic acidemias (pyruvate transporter, pyruvate carboxylase, pyruvate dehydrogenase system and Krebs cycle defects), mitochondrial respiratory chain disorders, mitochondrial transporters of energetic and other indispensable molecules,, coenzyme Q biosynthesis, FA oxidation and ketone body defects. A large and growing group of already > 110 disorders involves mitochondrial machinery (Frazier et al. 2017).
2.3 *Cytoplasmic energy defects* include glycolysis, glycogen metabolism, gluconeogenesis, hyperinsulinism, creatine metabolism disorders and finally inborn errors of the pentose phosphate pathways.

The brain accounts for 20% of an adult’s energy expenditure at rest and more than 50% in a child (Sokoloff 1960). Neurons expend 70–80% of total energy (the remaining portion used by glia) and the great majority (80%) is utilised to fuel neuronal channels. We could then hypothesise that energy defects in the brain tend to behave as “synaptopathies”, thereby encompassing those symptoms within the spectrum of synaptic disorders (Tristán-Noguero and García-Cazorla 2018, this issue).

Energy compartmentalisation between neurons (lactate use, pentose pathway, oxidative phosphorylation) and glia (mostly glycolytic), and even inside different compartments of a single neuron, has been now largely described (Oyarzábal and Marín-Valencia n.d., this issue; Camandola and Mattson 2017). Fuel molecules such as ATP and lactate also have signalling roles promoting synaptic plasticity. Energy use for axonal transport and nerve conduction is also compartmentalised. Electrically active axons not only rely on glycolysis but also need the supply of lactate provided by oligodendrocytes (that surround axons covering them with myelin) (Trevisiol et al. 2017). Glucose is the obligatory fuel for adult brain, but lactate produced from glucose by astrocytes within brain during activation has been proposed to serve as neuronal fuel. This simple and seductive hypothesis is far from being proven (Dienel 2017). Whatever the source of ATP may be, ATP consumption is greatly increased in demyelinated axons due to the redistribution of ion channels along the length of the denuded axon (Alizadeh et al. 2015; Salzer 2015) and decreasing mitochondrial ATP production results in free radical production leading to lipid peroxidation and severe physico-chemical modifications of cellular and organelle membranes (Dobretsov et al. 1977).

*Given the vulnerability of energy homeostasis in the brain, most neurological disorders, and in particular, neurodegenerative diseases are necessarily linked to disturbances in energy metabolism.*

3) Complex molecules

This expanding group encompasses diseases that disturb the metabolism of complex molecules that are not or poorly water soluble or diffusible. In general, these defects have no easily identified metabolic markers and diagnosis is primarily based on molecular techniques (NGS, WES). The main chemical categories of such complex molecules encompass the glycogen, the sphingolipids, the phospholipids, the cholesterol and bile acids, the glycosaminoglycans, oligosaccharides and glycolipids and the nucleic acids. Like the small molecules, there are two subcategories in complex molecule disorders defined by whether the phenotype primarily results from an accumulation or a deficiency.

These complex metabolic processes involve all organelles (mitochondria, lysosomes, peroxisomes, endoplasmic reticulum, Golgi apparatus, lipid droplets and the synaptic vesicle) and are highly regulated. Cellular membranes are formed from a chemically diverse set of lipids present in various amounts and proportions. Membrane lipids fulfil many functions including membrane structural components, energy and heat sources, signalling molecules, protein recruitment platforms and substrates for post-translational protein–lipid modification (Harayama and Riezman 2018). There are two subcategories in complex molecule disorders defined by whether the phenotype primarily results from an accumulation or a deficiency.

3.1 *Accumulation are linked to catabolism defects* leading to storage of a visible accumulated compound (classical lysosome defects like sphingolipidoses or mucopolysaccharidoses) in which signs and symptoms primarily result from the abnormal accumulation of compound(s) proximal to the block and potentially reverse as soon as the accumulation is removed.
3.2 Deficiency are linked to synthesis and remodelling of these complex molecules. They share some characteristics with the vast new group of processing, trafficking and quality control disorders that involve also protein metabolism. They may interfere with embryo and foetal development with neurodevelopmental disruptions, have a congenital presentation and present as birth defects. Symptoms are permanent, progressive, and independent of intercurrent events. This rapidly expanding group encompasses classic organelle disorders (lysosome and peroxisome defects), carbohydrate-deficient glycoprotein (CDG) syndromes and the synthesis defects of all kind of complex molecules biochemical categories.

All defects affecting systems involved in intracellular vesiculation, trafficking, processing of complex molecules and quality control processes (like protein folding and autophagy) recently discovered using the NGS technique belong to this category (Sprecher et al. 2005) (Hirst et al. 2015) (Stockler et al. 2014). This is also the case with the synaptic vesicle (SV) cycle (Cortès-Saladelafont et al. 2018, this issue) and congenital disorders of autophagy like SENDA or Vici syndrome (Ebrahimi-Fakhari et al. 2016).

The neurological manifestations are diverse, complex, with a predominance of motor symptoms (such as spastic paraparesis, ataxia and movement disorders), are often progressive and may be associated with extra-neurological signs. Trafficking, autophagy and quality control processes are major issues in regard to neurochemical mechanisms. These diseases are leading the way to a new neurology that connects metabolism, neuroscience and clinical neurology.

4) Finally nucleic acid disorders are much more than the classic purine and pyrimidine defects when we include cytoplasmic and mitochondrial tRNA synthetases defects, (Wallen and Antonellis 2013; Smits et al. 2010); ribosomopathies (Mills and Green 2017); diseases affecting mechanisms of DNA/RNA damage reparation and those involved in DNA methylation (DNAm) such as in CHARGE and Kabuki syndromes (Butcher et al. 2017).

## Part II. Integration of neurology, metabolism and cell biology

### The amazing evolution of neuroscience in recent years

The twenty-first century, with only 18 years of history, has already changed neuroscience thanks to significant new discoveries and techniques. On a global level, the study of the brain has moved to a system-neuroscience/system biology approach (Südhof 2017). The European initiative “Human Brain Project” (http://www.humanbrainproject.eu/en/) strives to accelerate neuroscience and brain-related medicine through the strategic alignment of scientific research programmes. It is precisely here, where metabolism and biochemical pathways, so under-represented in neuroscience thus far, can be of precious help. In clinical neurology, disorders are studied in function of the main sign or neurological syndrome, which is the traditional approach in medicine. Combining this symptom-based classification with the pathophysiological mechanisms (Table 1S, Table 2) is not always easy and rarely integrated in the practical procedure of neurologists.

**Table 2:** Spastic paraparesis and their corresponding cell biology mechanisms and metabolic impairment (cellular neurometabolism approach)

Predominant neurological syndrome	Gene	Disease, other symptoms	Biological role/location	Metabolic involvement	Reference
Spastic paraparesis Clinical signs: lower extremity spastic weakness that produces difficulty walking Symptoms may begin at any age. In infancy HSP may manifest as “toe walking” and mimic spastic diplegic cerebral palsy. HSP can be isolated or complex (with other symptoms). SPG (spastic gait) locus and the number refers to the OMIMno.	*KIF5a*	SPG10. Pure SP starting in childhood or at young adulthood. Sometimes distal sensory impairment	Axonal transport	Energy impairment, endosome/lysosome trafficking	Reid et al. 2002
	*KIF1A*	SPG30. Slowly progressive SP characterised by onset in the first or second decades.	Axonal transport	Energy impairment	Erlich et al. 2011
	*Spastin*	SPG4. Uncomplicated SP starting from childhood to adulthood	Defective mitochondria and vesicle transport, Endosomal tubule fission, trafficking	Energy impairment, complex molecule def, lysosome ultrastructural morphology	Starling et al. 2002
	*SPG7 (Paraplegin)*	SPG7. SP may associated cortical, cerebellar and optic nerve atrohpy	Quality control defect, ATP-dependent protease (mitochondrial inner membrane) that degrades misfolded proteins	Energy impairment, respiratory chain defects in mucle, UPR	Koppen et al. 2007
	*REEP1*	SPG31, distal motor neuropathy	Trafficking morphogenesis of the ER, mitochondrial function	Energy impairment, complex molecule def,	Beetz et al. 2008
	*ATL1*	SPG3A, early onset HSP, Hereditary Sensory Neuropathy Type 1D	Trafficking morphogenesis of the ER	Complex molecule defect	Abel et al. 2004
	KIAA0196, NIPA1, Spartin	SPG 8 (with ataxia), 6, 20 (early childhood, cerebellar signs)	Trafficking endosome morphogenesis and signalling	Complex molecule defect	De Matteis and Luini 2011
	*KIAA 1840* *Spactasin*	SPG11. Most common AR HSP. Also related with juvenile ALS and CMT	Autophagy impairment, lysosome reformation	Complex molecule defect	Bauer et al. 2009
	*ZFYVE26*	SPG15. Second most common AR HSP. Complex, can associate epilepsy and dementia	Autophagy impairment, autophagosome maturation, lysosome reformation	Complex molecule defect	Hanein et al. 2008
	*TECPR2*	SPG49. Complicated, developmental delay, dysmorphic features, neurodegenerative disease	Autophagy impairment, autophagosome formation	Complex molecule defect	Oz-Levi et al. 2012
	IEM with predominant spastic paraparesis	**-**Complex lipid defects: such as *DDHD1, DDHD2, CYP2U1, NTE, FAH2, GBA2, B4GALNT1, PLA2G6* **-**Other complex molecule defects and in particular those leading to leukodystrophy (ADL, lysosomal…) -Small molecule diseases: arginase deficiency, HHH, Homocysteine remethylation defects, neurotransmitter defects			
	Spastic paraparesis General comments	We have included in this table only a small selection of genes. Metabolic abnormalities in both, neurogenetic and classic IEM include: **-**Complex molecule defects including trafficking, autophagy, quality control processes (UPR), complex lipid metabolism and remodelling are the most frequent mechanisms in HSP. -Energy defect including defects of ATP production and mitochondrial transport. -Interestingly some IEM of small molecules (mainly intoxication diseases) are also a cause of HSP. Further studies on the pathophysiology of these particular IEM would probably lead to defects related to trafficking and quality control mechanisms.			

This table contains diseases that are representative of well-defined cell biology mechanisms which can be “easily” linked to pathophysiological categories described in IEM. This is not an exhaustive list of genes related to spastic paraparesis and does not describe in detail the clinical features of every disease

Abbreviations: *ADL*, adrenoleukodystrophy; *ALS*, amiotrophic lateral sclerosis (progressive muscle weakness and paralysis by motor neuron degeneration); *CMT*, Charcot-Marie-Tooth; *ER*, endoplasmic reticulum; *HSP*, hereditary spastic paraparesis; *SP*, spastic paraparesis; *UPR*, unfolded protein response

### Metabolism is a major regulator of brain functions

The concept of “molecular machines” was introduced by Alberts about two decades ago to describe large assemblies of biomolecules that are specialised to perform particular cellular functions (Alberts 1998). Despite metabolism strongly orchestrates the regulation of neuronal functions, the “metabolic” approach of brain disorders has been mostly neglected so far. For instance, energy metabolism participates in the intricate organisation of postsynaptic density which is a complex signalling structure involving many proteins related to neuronal plasticity and learning, and is an important niche in monogenic causes of intellectual disability (Camandola and Mattson 2017; Bayès n.d., this issue). Other examples such as vesicular glycolysis, which is used for fast axonal transport (Zala et al. 2013), P4-ATPases which uses the energy from ATP hydrolysis to transport specific lipids across membranes (Martín-Hernández et al. 2016), and complex networks of interactions between SNARE proteins and lipids (mostly phospholipids) that regulate synaptic vesicle trafficking, docking and therefore neurotransmitter release (Darios et al. 2009) are processes that rely on pure biochemistry but are linked to classical “neurogenetic” disorders.

### Main cell types, biological functions and metabolites in the brain (Figs. S1, S2)

Neurons and glia (astrocytes, oligodendrocytes and microglia) are the two major cell types. Neurons have many phenotypes that reflect different structures, functions, and metabolic signatures but there is still no consensus on how neuron types should be defined. The way that a neuron communicates determines its identity, which in turn relies on functions such as synaptic plasticity and neuronal excitability, the basis of learning and behaviour. These neurobiological functions are prone to be damaged in almost any kind of neurological disease. Not only metabolites, the “drivers” of neurobiological functions, behave mainly as structural molecules, signalling molecules and fuels but the same metabolite may also have more than one function which can also change over time. For example, dopamine is a neurotrophic factor in early development and a neurotransmitter later on. Additionally, metabolites in the brain are highly compartmentalised which is defined as the presence of different neuronal compartments (such as soma, axon, synaptic terminal, each with specific metabolic properties) and more than one distinct pool of a given metabolite. These separate pools of a metabolite are not in rapid equilibrium with each other but maintain their own integrity and turnover rates. At the same time, a particular molecule may have a function in only one anatomic structure or cell type but not elsewhere. For instance, lactate is important for the establishment of long-term memories acting in the neurons of the hippocampus (Suzuki et al. 2017).

### A functional approach of diseases: “cellular neurometabolism”

**Fig. 1:**
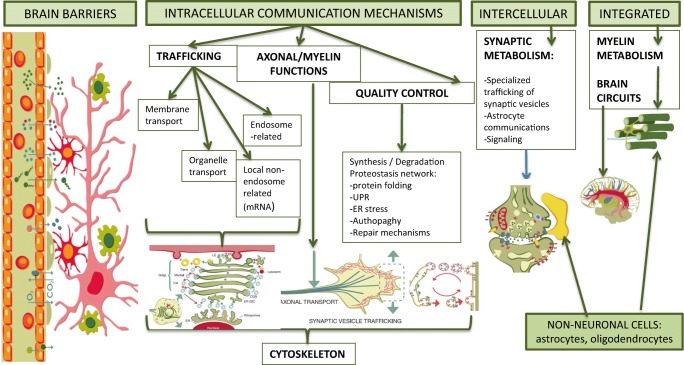
Cellular Neurometabolism: compartmentalised signalling and metabolism in the brain. Schematic representation of compartments that illustrate microenvironments of biological and metabolic functions. Global overview of the “cellular neurometabolism” approach

Neurons have polarised morphologies. They are very well compartmentalised into pre- and postsynaptic regions, synapses, dendrites, somas and axons, which are essential for their functions. They are the largest known cells, with process lengths ranging from millimetres to centimetres and metres (axons). This combination of specialised morphology with extreme length imposes a need for signals to be actively transferred between neuronal compartments (Wiegert et al. 2007; Terenzio et al. 2017). To integrate information within and between sub-neuronal compartments, neurons need sophisticated transfer mechanisms that ensure necessary molecules are available or generated in the right place at the right time. Metabolites participate in all these processes in a compartmentalised way. Categories of IEM as initially classified could also be related (at least partially) to this “neuronal microanatomic and functional” point of view. The following are models of compartmentalised signalling and metabolism (Fig. 1):

1. Brain barriers

In order to protect the brain from fluctuations in the blood composition, the blood–brain barrier (BBB) and the blood–cerebrospinal fluid barrier (BCSFB) regulate the exchanges of molecules between blood and cerebral fluids. These barriers, made of a variety of complex lipids, limit the free diffusion of metabolites between blood and brain fluids, and selectively transport essential nutrients (AA, lipids), ions and signalling molecules. Only six IEMs related to BBB-specific defects involving the transport of glucose, monocarboxylates, AA and lipids have been described (Fig. S2, Table 1) and only one in the BCSFB, the FOLR1 defect, (Steinfeld et al. 2009). However, secondary disturbances of essential AA transport is commonly observed in case of an elevation of one specific neutral AA using the L-type AA carrier like phenylalanine or leucine in PKU and MSUD, respectively. This observation has been used to treat PKU (Matalon et al. 2006)***.***

*Brain barrier defects belong to the category of small molecule defects (both essential and non-essential molecules) and are potentially treatable although they can present different degrees of clinical response.*

(2) Intracellular communication

Organelles, proteins and RNAs are actively transported to synaptic terminals for the remodelling of pre-existing neuronal connections and formation of new ones (Wojnacki and Galli 2016). Mechanisms of intracellular communication can be divided into the following different categories:

1. Trafficking

**Fig. 2:**
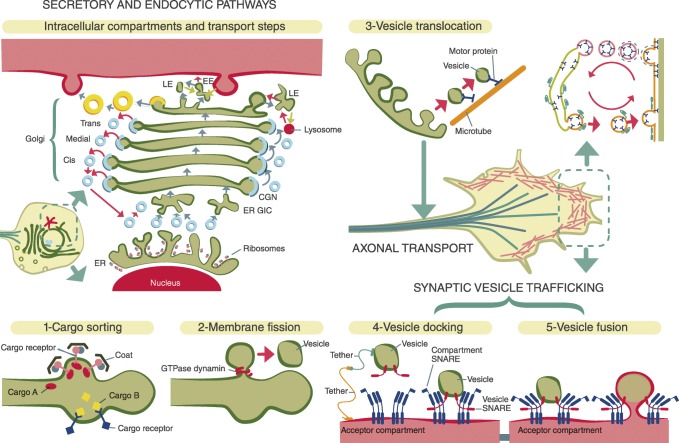
Trafficking: compartmentalization, cell biology and metabolic functions. The secretory and endocytic pathways: the transport of synthesised proteins starts from the ER. After folding, proteins are sorted into budding vesicles generated through the coat protein complex COPII. Vesicles move to ER-Golgi intermediate compartment and the cargos are transported to the Golgi complex, where proteins return to the ER (depending on coat protein complex I: COPI). At the TGN cargos are packaged in vesicles which carry them to their final destinations (i.e: lysosomes, plasma membrane, secretory granues…). Membrane proteins may undergo clathrin dependent and independent endocytosis. Vesicles can be transported (3-vesicle translocation) through specific mechanisms along the axon (see Fig. 5), where they undergo cycles of 4-vesicle docking (SNARE proteins) and 5-fusion with the plasma membrane and re-endocytosis. One example of this functions is represented by the synaptic vesicle cycle, at the presynaptic terminal. Abbreviations: ER, endoplasmic reticulum; ERGIC, ER-Golgi intermediate compartment; CGN, cis-Golgi Network; TGN, trans Golgi Network; LE, later endosome; EE, early endosome; Yellow vesicles, clathrin-coat; Small blue vesicles, COPII-coat

Membrane traffic is a highly regulated process that ensures the communication between membrane-bound compartments while maintaining their specific protein and lipid composition (De Matteis and Luini 2011). Although the various membrane compartments (endoplasmic reticulum (ER), Golgi, endosomes, vesicles, organelles…) use a different repertoire of proteins and complex lipids, the basic principles are similar and divided into four main steps that travel from cargo sorting to fusion with the acceptor compartment (Fig. 2). Additionally there is an endosome-free route through mRNA binding proteins for local translation (Boulay et al. 2017; Sakers et al. 2017). Membrane and endosome-related trafficking can lead to a great variety of neurological diseases (Table S2). As a rule, spastic paraparesis is mostly related to defects localised at ER, endosomes and lysosomes(Table 2). Peripheral neuropathies and in particular, diverse subtypes of Charcot-Marie-Tooth, as well as muscle dystrophies, are more likely to have their localisations at the plasma membrane. Trafficking defects between the ER and the Golgi are related to complex neurological and extra-neurological syndromes (Table S1). Finally, and localised at the presynaptic terminal, synaptic vesicle disorders are in great majority trafficking diseases that produce synaptopathies (spectrum epilepsy, intellectual disability, neuropsychiatric signs, movement disorders) (Cortès-Saladelafont et al. 2016).

*Disorders of trafficking belong to the category of complex molecule defects. Several glycosylation defects (COG1, 7, 8) are related to trafficking alterations between the ER and the Golgi complex. Lysosome biogenesis defects (Hermansky-Pudlak syndromes; type 7 and 8) and several phosphoinositide-phosphatase-producing myopathies, Lowe syndrome and CMT types 4B1 and 4B2 also belong to this category.*

b.2 Cytoskeleton, axonal and myelin functions

**Fig. 3:**
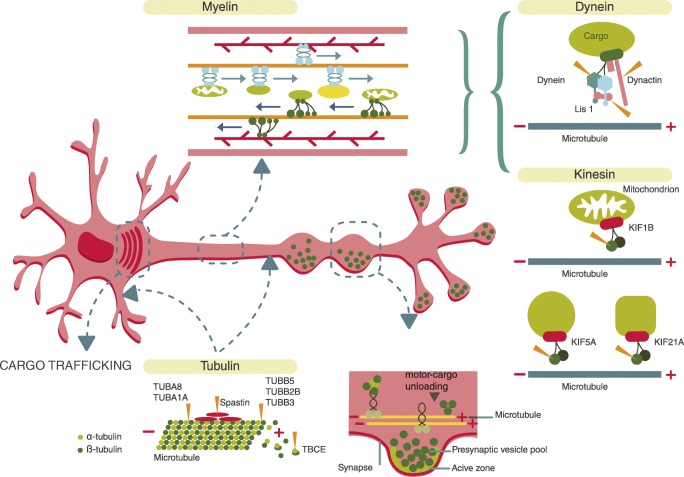
Cytoskeleton and axonal structure and functions. Impaired neuronal transport can be caused by disruption of the microtubule network. Tubulin (bottom left in the figure) is a cytoskeleton component in both dendrites and the axon and is composed by alpha and beta subunits. Different mutant proteins (TUBB subtypes including TBCE: tubulin chaperone) produce a repertoire of diseases ranging from cortical migration defects to motor neuropathies and spastic paraparesis (see Table 2). Axonal transport undergoes cargo release at the synaptic and presynaptic buttons. Neurons must be constantly transferring mitochondria, vesicles, other organelles and RNPs granules depending on the cells need. Transport is bidirectional along the axon where kinesin allows for anterograde transport, dynein provides retrograde transport. This is crucial for the proper distribution of proteins, transcripts and organelles. Adaptor proteins (such as dynactin and Lis 1 in the picture) mediate the specific binding between motor proteins and their cargo. Crosstalk between adaptors and other regulators is possible following the movement of either kinesin or dynein. Members of the kinesin family (different KIF subtypes in the picture) mediate the transport of late endosomes, lysosomes synaptic vesicle precursors and dense core vesicles. Some diseases related to these biological processes can be found in Table 2

These functions are related to transport via motor proteins in different compartments: soma, dendrites and axons. They are also linked to nerve impulse propagation thanks to the collaboration between the axon and myelin. Proteins of the cytoskeleton play a central role in the creation and maintenance of cell shapes. They provide structural organisation helping to establish metabolic compartments and serve as tracks for intracellular transport (Fig. 3). Motor proteins, including the myosin, dynein and kinesin families of proteins, are responsible for the anterograde and retrograde transport of cargo. In general, myosins are actin-dependent motors, whereas dyneins and kinesins are microtubule-dependent motors (Namba et al. 2015). There are different mechanisms by which cargo (including organelles, mRNA and receptors) are selectively transported to the appropriate subcellular region by motor proteins including protein kinase regulation (Gibbs et al. 2015). Specific diseases are linked to deficits in these mechanisms and motor proteins themselves (Fig. 3, Table S1). Most of them are motor diseases (Tables 1 and S1) (motor neuropathies, CMT2, HSP, ALS, SMA, CFEOM: congenital fibrosis of extraocular movements) and cortical migration abnormalities (Table S1). Organelles (mitochondria, lysosomes, the synaptic vesicle, lipid droplets), autophagosomes, neurotrophin receptors and mRNA, are also transported through the axon by a set of motor proteins and their regulators that are different in every organelle and structure and also have their own diseases (Fig. 3). Myelin from oligodendrocytes covers almost the entire axon in order to provide a fast, low energy-consuming conduction. Myelin composition and related disorders are very numerous and go beyond the scope of this article. Some important metabolic aspects of cytoskeleton, axon and myelin are highlighted below. Disorders of mitochondrial transport exhibit abnormal mitochondrial shape and motility and in some cases decreased oxidative phosphorylation function. Moreover impaired mitochondrial transport has an impact at the presynaptic terminal due to the energy requirements for neurotransmission.

Myelinating glia have been found to be critical for transferring energy metabolites (i.e. lactate) to axons through monocarboxylate transporters, which are present on the inner membrane of the glial sheath and on the axon. However, the role of lactate as a main source of energy to neurons remains severely debated (Dienel 2017) and MCT1 deficiency does not give rise to demyelination (van Hasselt et al. 2014). While myelination markedly reduces the energy requirements for action potential propagation, energy is still required for axonal transport, metabolism and action potential regeneration at nodes (Salzer 2015).

Alterations in membrane potential and synaptic homeostasis lead to severe epilepsies and other progressive neurological features. This is the case of KIF5A deficiency (Rydzanicz et al. 2017) and TRAK1 mutations (Barel et al. 2017) (Table S1). On the other hand, myelin metabolic diseases are strongly related to complex lipid synthesis and remodelling defects but also particularly to those diseases where the enzyme/transporter/metabolic defect is primarily localised at the oligodendrocytes (metabolic leukodystrophies).

b.3 Quality control mechanisms

The balance between synthesis and degradation in neurons is of utmost importance because there are long distances from the neuronal cell body, where most proteins are synthesised to their place of action. The slow rates of axonal transport are not ideal for replacing misfolded or dysfunctional synaptic proteins on demand. Due to the sustained nature of neurotransmission, the synaptic proteome is prone to protein misfolding. To maintain their specialised structure and function, neurons possess dedicated synapse-specific proteostasis machinery localised to pre- and postsynaptic compartments (Gorenberg and Chandra 2017). Alterations in this network, in particular, disturbances in ER functions trigger abnormal protein aggregation. In turn, ER stress triggers a signalling reaction known as UPR (unfolded protein response) that is also related to synaptic function (Hetz and Saxena 2017). Alterations in the proteostasis network have been related to a great diversity of neurodegenerative diseases (Table S1).

Disorders of quality control mechanisms involve both, small and complex molecules. Protein-folding diseases are related to chaperone defects such as DNAJC12 and DNAJC6, involved in neurotransmitter disturbances and a spectrum of symptoms including early Parkinsonism (and hyperphenylalaninemia in the case of DNAJC12 (Anikster et al. 2017)). A recent study shows that ER stress is involved in the pathophysiology of homocystinuria (Martínez-Pizarro et al. 2016) leading to the hypothesis that other intoxication defects could potentially be related to ER stress and consequently, UPR.

Autophagy defects also belong to this category beside monogenic mitophagy diseases (DJ-1, PINK1 and Parkin) related to Parkinson’s disease. Interestingly, a neurotransmitter defect, SSADH, triggers impaired mitophagy at the postsynaptic level through the mTOR pathway due to the high concentration of GABA at the synaptic cleft (Vogel et al. 2016). Finally, metabolism defects in metabolic repair are exemplified by NAXE deficiency. NAXE catalyses the epimerization of NAD(P)HX, thereby avoiding the accumulation of toxic metabolites (Kremer et al. 2016).

(3) *Intercellular communication* takes place at the synaptic level and is composed of a presynaptic neuron, a postsynaptic neuron and a astrocyte (tripartite synapse). Mechanisms of communication here could be grouped under the name of “synaptic metabolism” and are explained in detail elsewhere (Tristán-Noguero and García-Cazorla 2018, in this issue).
(4) *Integrated communication* of the cellular and subcellular structures and functions are connected throughout the brain by means of axons, myelin and the formation of circuits, thereby creating an entire system. These functions are very complex and go beyond the scope of this article.

## Conclusions and future directions

Brain metabolism, which has been largely disregarded in the traditional approach of neurological diseases, is a major clue and probably the next imminent “revolution” in neurology and neuroscience. Indeed metabolism is a universal language, an adaptive biological response. Therefore, any kind of disorder can potentially be observed and “explained” under this metabolic perspective. In the last recent years, new categories of IEMs have demonstrated the necessity of integrating both fields, cell biology and classic metabolism, in order to better understand mechanisms of disease. This approach leads to a redesign of the previous IEM classification as well as to an effort to understand brain disorders under a different angle. This article is an attempt to move toward this direction. However, the complexity and novelty of this essay make it provisional and under constant revision. As we go further in the description of the pathophysiological mechanisms of a single disease, we discover the many different pathways that can be involved. In the future, medical doctors would need to be able to combine symptoms with map diseases (made up of pathophysiological categories) and treatment possibilities. In this context, metabolomics and lipidomics stand out among omics as they study the end products of cellular processes and therefore are more likely to be representative of clinical phenotypes than genetic variants or changes in gene expression (Wevers and Blau 2018). By now, this is a big challenge that has just started.

## Electronic supplementary material

ESM 1 (DOCX 82 kb)

Fig. S1 Main cell types, biological functions and metabolites in the brain. Cell morphology and typology: the neocortex comprises two major cell types: neurons and glia. Neurons are the signalling cells of the nervous system, and glia perform myriad functions to support the function of neurons. Neurons can be further subdivided into excitatory projection neurons (glutamatergic)and inhibitory interneurons (Gabaergics). Only some examples of GABAergic cells are provided. Micro and Macro-communication refers to networks of brain cell communication at the synaptic level and in the organisation or brain circuitries. Further explanations about the main synaptic biological functions and the role of metabolites in the brain are provided throughout the text. (JPG 90 kb)

Fig. S2 Blood-brain-barrier (composed by endothelial cells and astrocytes) and transporters related to IEM. NEUROMETABOLIC DISEASES LINKED TO BLOOD BRAIN BARRIER (BBB) DEFECTS: Of the 14 **GLUTs** transporters, only GLUT1 and GLUT-10 are related with brain diseases. Lactate: - taken up from circulation via MCT1(expressed in endothelial cells, astrocytes and oligodendrocytes); −produced by glycolytic astrocytes and oligodendrocytes; −secreted via MCT1 and MCT4. The neuron-specific MCT2 mediates lactate uptake into the neurons. Of the 22 amino acid transporters at the endothelial cells in the BBB, only 2 are related with diseases. BCAA (branched chain amino acid) defect mimicks BCDHK (branched chain dehydrogenase kinase) defect; SLC6a14 transporter is expressed in astrocytes and release serine trom the astrocytic synthesis of serine and is also expressed at the endothelial cells of the BBB although with poor permeability. Among the Lipid transporters in the BBB (lipoprotein receptors, ABC transporters and fatty acid transporters), only one recently described defect, Mfsd2a mutations, is related with human disease.). The brain does not synthesise DHA, which is imported across the BBB through the Major Facilitator Superfamily Domain 2a (Mfsd2a). Mfsd2a transports DHA (docohexanoic acid) as well as other fatty acids in the form of lysophosphatidylcholine (LPC). (JPG 67 kb)

## Abbreviations

IEM: Inborn error of metabolis
