# Supplementary material for: Cellular neurometabolism: a tentative to connect cell biology and metabolism in neurology
Source: J Inherit Metab Dis. 2018 Jul 16;41(6):1043–54. doi: 10.1007/s10545-018-0226-8 (PMC6326994; doi:10.1007/s10545-018-0226-8)
Supplement: Supplementary file 1 — (DOCX 82 kb) [file 10545_2018_226_MOESM1_ESM.docx]

| **PREDOMINANT NEUROLOGICAL SYNDROME** | **GENE** | **DISEASE, OTHER SYMPTOMS** | **BIOLOGICAL ROLE/LOCATION** | | **METABOLIC**  **involvement** | | **REFERENCE** |
| --- | --- | --- | --- | --- | --- | --- | --- |
| ***Motor neuron disease***  Clinical signs :  Focal motor weakness, usually in the distal limbs but sometimes in bulbar muscles.  In general, progression to paralyze all skeletal muscles.  In some cases dementia can be associated  ALS (in adults) and SMA (in children) are the most common clinical forms of motor neuron diseases. | *ALS2-Alsin* | ALS | **Axonal transport** Cargo protein. Activation of Rab5 for endosome dynamics | | Trafficking, autophagy | | Devon et al, 2006 |
|  | *VAPB* | ALS, PMA, SMA | **Defective mitochondria**  **and vesicle transport**  **UPR** | | Energy impairment  Complex molecule def | | Aliaga et al, 2013 |
|  | *Dynactin-1* | ALS, PMA, Perry syndrome | **Axonal transport** **and vesicle trafficking**, impaired signaling endosome trafficking | | Energy impairment  Complex molecule def:  Trafficking  Autophagy | | Munch 2004 |
|  | *Dctn1*  (Dynactin complex component) | ALS; vocal fold paralysis, facial and distal weakness | **Retrograde transport**; mutant protein aggregates | | Energy impairment, autophagy, ERS, UPR | | Levy et al, 2006 |
|  | *FUS* | ALS | **Defective mitochondria transport**,  **defective RNA processing,** aberrant microtubule acetylation | | Energy impairment  RNA processing,  UPR | | Yang et al, 2010 |
|  | *Spatacsin* | ALS, SPG11 | **Axonal transport**  Reduced tubulin acetylation, reduced anterograde vesicle transport | | Energy impairment  Complex molecule def  Acetylation | | Orlacchio, 2011 |
|  | *SOD*  (Superoxid dismutase-1) | ALS | **Defective mitochondria transport** , microtubule stability, modulation of motor proteins via p38MAP kinase | | Energy impairment, UPR | | Deng, 1999 |
|  | *TARDBP* | ALS, FTD | **Defective mitochondria transport**, **defective RNA processing,** impaired microtubule acetylation | | Energy impairment  RNA metabolism (processing) | | Benajiba, 2009 |
|  | *TUBA4A* | ALS | Destabilization of microtubules, **general transport defect?** | | Energy impairment, trafficking | | Smith, 2014 |
|  | *FIG4* | ALS | **Autophagy impairment**  P62 accumulation | | Phosphoinositide phosphatase, Complex molecule def, | | Chow 2009 |
|  | *SMN1* | SMA | **Axonal transport**  Reduced transport of specific mRNA | | RNA metabolism or translation | | Muller, 1992 |
|  | *AR*  (Androgen receptor) | Kennedy disease (X-linked SBMA) | **Axonal Transport**  Mutant protein blocks axonal transport | | Energy impairment | | Butler et al, 1998 |
|  | **IEM with motor neuron disease as predominant clinical symtom** | **-Complex lipid defects :** PNPLA6, HSAN1  **-Other complex molecule defects** such as: Farber disease (lysosomal disorder, ASAH1 in its late onset presentation), GM1, GM2, Krabbe, Adult polyglucosan body disease, peroxisomal defects.  -**Energy defects**: mitochondrial (OXPHOS; mitDNA depletion), TK2 defect, biotinidase deficiency | | | | | |
|  | **MOTOR NEURON**  **DISEASE**  **General comments** | Biological processes and pathways in ALS and SMA are linked to neurodegeneration.  We have included in this table only a small selection of genes.  **Metabolic abnormalities in both, neurogenetic and classic IEM include:**  **-Mitochondrial dysfunction** (SOD is the most representative, but many others are included in the list above)  **-RNA metabolism** (other than those in the table: *TDP-43, ELP3, ANG, SMI*, Senataxin, Ataxin2, *C9orf72, hnRNPA1*). They regulate functions such as transcription, splicing, DNA repair and RNA transport.  **-Quality control mechanisms** and in particular UPR (*TD43, FUS, SOD1, C9orf72, UBQLN1-2, SOSTM1*  -**Trafficking, which is related to lysosomal and complex lipid** **metabolism** (synthesis and remodeling defects) | | | | | |
| ***Peripheral neuropathy***  Clinical signs:  Distal muscle weakness, often involving lower legs and feet, atrophy, diminished or absent deep muscle reflexes, abnormal gait (steppage), foot deformities (pes cavus) are frequent  CMT defines the historic description of neurogenetic neuropathy. IEM, (some of them treatable), produce similar symptoms. Clinically they can be axonal (A)(motor conductions affected), demyelinating (D)(loss of surrounding myelin), and sensorial (S)(small fiber involvement). | *Kif1b* | CMT2a1  axonal | **Axonal Transport**  Transport of SV precursors and mitochondria | Energy impairment, trafficking | | Zhao et al, 2001 | |
|  | *DYNC1H1* (dynein) | CMT2  axonal | **Axonal transport** (retrograde): vesicular and organelle | Energy impairment  Complex molecule def. | | Eschbach, 2013 | |
|  | *Mfn2:* mitofusin 2 | CMT2a  axonal | **Axonal Transport Defective mitochondria transport** | Energy impairment | | Calvo et al, 2009 | |
|  | *Kinesin 5A* | CMT2 (axonal)  Other phenotypes: myoclonus intractable, neonatal,  HSP | **Axonal transport** vesicular and organelle | Energy impairment  Complex molecule def. | | Crimella et al, 2012 | |
|  | *KIF1B beta* | CMT2 (axonal) | **Axonal transport Defective mitochondria and vesicular transport** | Energy impairment  Complex molecule def. | | Drew et al, 2015 | |
|  | *GDAP1* | Axonal neuropathy | **Axonal transport**  **Defective mitochondria transport**  Interaction cytoskeleton and mitochondria | Energy impairment | | Baxter et al, 2002 | |
|  | *TUBB3* | Late-onset axonal sensorimotor neuropathy | **Neurogenesis axon guidance and maintenance** |  | | Tischfield, 2010 | |
|  | *DNM2* | CMT  Intermediate, axonal, hypomyelinating (letal congenital contractures) | **Trafficking**  Defective vesicle bending or fission | Complex molecule def. | | Claeys et al, 2009 | |
|  | *FIG4, MTMR13, MTMR2* | Different subtypes of CMT | **Trafficking** | Phosphoinositide phosphatase deficiency | | De Matteis, 2011 | |
|  | *FGD4, RAB7A* | CMT 4H, 4C demyelinating | **Trafficking** | GTPase defects | | De Matteis, 2011 | |
|  | **IEM with predominant**  **peripheral neuropathy** | -**Energy defects:** mitochondrial (OXPHOS, PDH) (A), MNGIE (D); beta-oxidation (LCHAD, TF) (A)  **-Complex lipids defects:** *HSAN1, PLA2G6, CYP2U1, GBA2, B4GALNT, NTE* (A, D, S)  **-Other complex molecule defects:** CDG syndromes, (A, D) Krabbe (D), peroxisomal defects (such as Refsum disease and ADL, AMN)(D), CTX (A, D)  -**Small molecule defects:** homocysteine remethylation defects (D), serine deficiency, porphyrias (A, S) | | | | | |
|  | **PERIPHERAL NEUROPATHY**  **General comments** | We have included in this table only a small selection of genes.  **Metabolic abnormalities in both, neurogenetic and classic IEM include:**  **-Energy defects** related to mitochondrial functions (ATP production) and transport.  **-Trafficking, which is related to lysosomal and complex lipid** **metabolism** (synthesis and remodeling defects). | | | | | |
| ***Muscle disorders***  Clinical signs:  Muscle diseases are characterized by weakness, (MW)and/or exercise intolerance, (EI)and/or associated cardiomyopathy (CMNO) | *KIF21a* | CFEOM: congenital fibrosis of the extraocular muscles | **Axonal transport** |  | | Yamada et al, 2003 | |
|  | *TUBB3* | CFEOM |  |  | | Poirler, 2010 | |
|  | *MTMR14* | Centronuclear myopathy | **Trafficking, Autophagy** | Phosphatidylinositol 3,5-bisphosphate (PI(3,5)P2) | | Tosch et al, 2006 | |
|  | *MTM1* | X-linked myotubular myopathy | **Trafficking** | Phosphatidylinositol 3-kinase defect | | Blondeau et al, 2000 | |
|  | *DYSF* | Muscular dystrophy | **Trafficking**  Vesicle fusion defect | Complex molecule defect  Membrane remodelling | | Bansal et al, 2003 | |
|  | *CAV3* | Muscular dystrophy | **Trafficking**  Vesicle coat defect | Complex molecule defect | | Galbiati et al, 1999 | |
|  | *DNM2* | Myopathy, centronuclear myopathy | **Trafficking**  Defective vesicle bending or fission | Complex molecule def | | Bitoun et al, 2007 | |
|  | *TANGO2* | Recurrent metabolic crisis with rhabdomyolysis | **Transport and Golgi organization 2 homolog** | Energy  Impairement ?  Trafficking | | Lalani et al, 2016 | |
|  | *TRAPPC11* | Limb-girdle muscular dystrophy 2S | **Trafficking**  Defective vesicle trafficking | Complex molecule def | | Borgehaussen et al, 2013 | |
|  | **IEM with predominant myopathies** | -**Energy defects:** mitochondrial defects,(MW,EI,CMNO) beta-oxidation such as ETFDH,(MW,CMNO) CPT2,(EI,MW,CMNO),FAD disorders (MW,EI,CMNO) primary carnitine deficiencies (MW,CMNO), muscle glycogenosis.(MW,EI,CMNO) Creatine defects.(MW); TANGO 2 (EI) mimics beta-oxidation disorders  **-Complex lipid defects:** *LIPIN1,(EI) CHKB, PNPLA2, ABHD5 (MW)* | | | | | |
|  | **MUSCLE DISORDERS**  **General comments** | Dystrophies, Channelopathies, Myasthenias and IEM are the main subtypes of muscle disorders. We have included in this table only a small selection of genes.  **Metabolic abnormalities in both, neurogenetic and classic IEM include:**  -**Trafficking, which is related to complex lipid remodeling and synthesis**  -**Energy defects,** most of them related to ATP production defects, and in CFEOM linked to axonal transport. | | | | | |
| *Ataxia*  Clinical signs:  Impairment of equilibrium and gait. Most associate basal ganglia, spinal cord, optic nerves, retina and peripheral nerve involvement  Genetic causes of inherited ataxia are very numerous. One of the most common subtype are SCA (spinocerebellar ataxia: slowly progressive cerebellar ataxia of the trunk and limbs, slowness of movements, impaired speech, nystagmus an tremor). Dysarthria, parkinsonian signs and dementia may appear later on | *SNX14* | SCA (AR) | **Autophagy impairment**  Autophagosome –lysosome fussion | Complex molecule defect | | Akizu et al, 2015 | |
|  | *ATG5* | SCA | **Autophagy impairment**  Autophagosome elongation | Complex molecule defect | | Kim et al, 2016 | |
|  | *SQSTM1/p62* | Ataxia, dystonia and gaze palsy, childhood onset neurodegeneration | **Autophagy impairment**  Cargo detection impairment | Complex molecule defect | | Haack, 2016 | |
|  | **IEM with predominant ataxia** | **-Energy defects:** mitochondrial, CoQ10 defects, PDH, GLUT-1 deficiency, mitochondrial ATP production and iron homeosthasis in Friedreich Ataxia.  **-Complex lipid defects:** PNPLA6; PLA2G6 and other causes of NBIA, it is also a frequent sign in diseases that produce HSP.  **-Other complex molecule defects:** Niemann-Pick C, GM2, Gaucher 3, CTX, Sialidosis, Mannosidosis, CLN, Refsum, CDG | | | | | |
|  | **ATAXIA**  **General comments** | DNA repeat expansions are a common cause of SCA. Ataxia is also strongly associated with many HSP types. We have included in this table only a few selection of genes related to the focus of this article.  **Metabolic abnormalities in both, neurogenetic and classic IEM include:**  **-Complex molecule defects including trafficking, autophagy, quality control processes (UPR), complex lipid metabolism and remodeling**  **-Energy defects** | | | | | |
| ***Parkinsonism***  Clinical signs:  Parkinsonism is a hypokinetic condition characterized by any combination of rest tremor, bradykinesia, rigidity, postural instability, freezing phenomena and flexed posture.  Many neurodegenerative diseases end up with parkinsonism signs. In chidren parkinsonism is very rare and normally associated to other neurological signs. | *Dynactin-1*  *(DCTN1)* | Perry syndrome* | **Axonal transport and vesicle trafficking** | Energy defect, complex molecule defect | | Farrer et al, 2009 | |
|  | *PINK1* | Early onset parkinsonism, slow progression, L-Dopa responsive. Psychiatric signs may be associated | **Autophagy , mitophagy** | Energy defect, mitochondrial protein with chaperone action  Neurotransmission (DA) defect | | Chen et al, 2013 | |
|  | *PARKIN* | Young onset Parkinson disease | **Autophagy,**  **mitophagy** | Energy defect  mitDNA repair  Neurotransmission (DA) defect | | Abbas et al, 1999 | |
|  | *DJ-1* | Early onset Parkinson disease, slow progression, good response to L-Dopa | **Mitochondrial dysfunction**  Increases ROS production, alteration of mitochondrial transmembrane potential | Mitochondrial dysfunction.  Neurotransmission (DA) defect | | Bonifati, 2003 | |
|  | *ATP13A2* | Kufor-Rakeb syndrome. PARK9. Juvenile pallido-pyramidal degeneration with L-Dopa responsive parkinsonism and dystonia, spasticity and dementia | **Membrane Trafficking**  Lysosomal type 5 P-type ATPase transmembrane active transporter. May associate NBIA | Lysosomal function.  Membrane trafficking. | | Bruggeman et al, 2010 | |
|  | SV defects and other synaptopathies |  | **Trafficking** | Complex molecule defects, lipid remodeling, protein-lipid, protein-protein interaction,  Defects of neurotransmission | | Cortès-Saladelafont, Tristan-Noguero, this issue | |
|  | **IEM with predominant parkinsonism** | -**Small molecule diseases:** neurotransmitter defects, metal disorders, Lesh-Nyhan  -**Complex lipid defects:** in particular those NBIA related  **-Complex molecule defects:** lysosomal defects such as CLN, Niemann-Pick C, GM, Gaucher, CTX  -**Energy defects:** POLG, other mitochondrial disorders | | | | | |
|  | **Parkinsonism**  **General messages** | We have included in this table only a small selection of genes.  **Metabolic abnormalities in both, neurogenetic and classic IEM include:**  **-Neurotransmission defects** (primary, due to neurotransmitter synthesis defects, or secondary in relation with loss of synaptic homeostasis, including mechanisms of neurodegeneration)  **-Complex molecule defects** including trafficking (strongly related to lysosomal diseases and complex lipid metabolism), and autophagy.  **-Energy metabolism** including ATP production and mitochondria homeostasis. | | | | | |
| ***Dystonia***  Clinical signs:  Movement disorder characterized by sustained or intermittent muscle contractions causing abnormal movements and postures  Dystonia is mainly a childhood neurological sign. The equivalent in adulthood is parkinsonism. Dystonia-parkinsonism complex refers to the presence of both signs in the same patient, which is a common feature in many diseases | Defects of the SV cycle at the presynaptic terminal  Other synaptic defects such as signaling | In general associated with other synaptopathy signs (epilepsy, ID, behavioural disturbances) | **Trafficking**  interactions of lipids and proteins with the SV and presynaptic membrane | Abnormal neurotransmission, quality control dysfunction , abnormal neurotransmission | | Cortès-Saladelafont, Tristan-Noguero, this issue | |
|  | Defects such as SCA3 (Machado-Joseph), *VPS35* (parkinsonism), *WDR45* (NBIA)  *TRAPC11*  ( muscle dystrophy) | Complex dystonias, associated to other neurological signs (mostly motor) | **Proteing degradation and UPR in SCA3 and WDR45**  **Trafficking (vesicular transport): VPS35, TRAPC11** | Complex molecule def. | |  | |
|  | **IEM with predominant**  **dystonia** | **-Energy defects:** mitochondrial disorders (such as Leigh syndrome), PDH, GLUT1, Thiamin transport defects, biotinidase, creatine defects  **-Complex molecule disorders:** lysosomal (Niemann-Pick C, GM, Gaucher, CLN), complex lipid defects (such as NBIA)  **-Small molecule defects:** neurotransmitter defects, intoxication disorders (homocystinuria, organic acidurias), metal disorders, purine disorders | | | | | |
|  | **DYSTONIA**  **General messages** | Metabolic dystonia is the most common cause of complex dystonia in childhood, in particular if we associate the disorders of the synaptic vesicle. Other pathophysiological categories are: defects of DNA repair, transcription and microtubules (cytoskeleton, such as tubulin ).  **All neurobiological and metabolic mechanisms described in this article can be at the basis of dystonic and other hyperkinetic movements.** | | | | | |
| ***Dementia***  Clinical signs:  set of symptoms that may include memory loss and difficulties with thinking, problem-solving or language. Progressive deterioration of cognitive functions | *C9orf72* | FTD | **Defective mitochondria transport** at the presynaptic level | Energy impairment | | Pearson et al, 2011 | |
|  | *TARDBP* | FTD, ALS | **Defective mitochondria transport** and mRNP granules, impaired microtubule acetylation | Energy impairment  Acetylation | | Benajiba, 2009 | |
|  | *CHMP2B* | FTD | Impaired endocytic **trafficking and autophagy.** .Disruption of endosome-lysosome fusion | Complex molecule defect, trafficking. | | Urwing, 2010 | |
|  | *Hungtingtin (HTT)* | Huntington disease, chorea, dementia | **Axonal transport** of BDNF is blocked | Energy impairment, BDNF is involved in AA metabolism and synaptic plasticity | | Gauthier et al, 2014 | |
|  | **IEM with predominant dementia and general messages** | In general, **complex molecule defects** at advanced stages of the disease.  They share mechanisms of neurodegeneration with these other neurogenetic conditions. Chronic hyperammonaemia due both to IEM and portocaval shunt can lead to dementia | | | | | |
| ***Multiple sclerosis***  Clinical signs:  Symptoms appear from the interruption of myelinated tracts in the CNS. Commonly limb weakness, sensory disturbance, monocular visual loss | *Kif1B* | Progressive demyelination | **Transport of mRNP in glia** |  | | Lyons et al 2009 | |
|  | *Kif21b* | Progressive demyelination | **Dendritic transport** | Neurotransmission | | Marszalek et al, 1999 | |
|  | **Disorders mimicking multiple sclerosis (MS) and general comments** | Pelizaeus-Merzbacher disease and spastic paraplegia type 2 are allelic X-linked disorders associated with defective myelination of the central nervous system and mutations in *PLP1*. Some energy defects, in particular those leading to nerve optic impairment (biotinidase deficiency, PDH).  Demyelination increases ATP consumption along axons. This can be related to the fact that high doses of biotine are an effective treatment for some forms of MS (Sedel et al, 2016) | | | | | |
| ***Leukodystrophies***  Are inherited disorders of white matter:  primary deficits in myelin development (hypomyelinating) , and demyelinating leuko- dystrophies, where myelin develops normally but subsequently undergoes progressive disruption. | *TUBB4A* Hypo-myelinating  Hypomyelinating leukodystrophy with atrophy of basal ganglia and cerebellum | Child: dystonia, nystagmus, mild cognitive deficit, ataxia and spasticity  Adolescent or adult: spastic ataxia or dystonia | **Cytoskeleton structure, neuronal guidance, migration** |  | | Miyatake et al, 2014 | |
|  | *PLP1.* Hypomyelinating | Pelizaeus–Merzbacher disease (HLD1): nystagmus, spasticity, dystonia. Adolescent: spastic ataxia | **ER stress and Golgi fragmentation** by inhibiting transfer of proteins from the Golgi back to the ER. | Quality control defects | | Numata et al, 2013 | |
|  | *POL3* related hypomyelinating conditions  *POL3A, B* | Dystonia, nystagmus, ataxia, spasticity, mild cognitive deficit, hypodontia and delayed or absent puberty | Polymerase III is a DNA-directed RNA polymerase that transcribes genes encoding ribosomal 5S RNA, tRNAs, small nuclear RNA and mitochondrial | RNA metabolism | | Bernard et al, 2012 | |
|  | **De-myelinating leukodys-trophies** | **The great majority of demyelinating leukodystrophies are caused by IEM** (complex molecule defects such as lysosomal disorders, peroxisomal, complex lipid defects and energy metabolism defects). Some neurogenetic non-metabolic are:  Progressive leukodystrophy with ovarian failure (LKENP ), Autosomal dominant adult-onset demyelinating leukodystrophy (LMNB1), Leukoencephalopathy with ataxia (LKPAT; CLCN2 gene), Leukoencephalopathy with vanishing white matter (VWMD ;  *EIF2B1*–5 gene), Megalencephalic leukoencephalopathy with subcortical cysts (MLC ; MLC1 and HEPACAM genes).  **Hypomyelination in IEM is a common feature in small molecule synthesis or transport defects.** | | | | | |
| ***Cortical migration defects***  This is a heterogeneous group characterized by an abnormal structure of the cerebral cortex  The clinical manifestations of these disorders vary considerably but developmental delay, epilepsy and motor problems are ferquent | *Lis1* | Lysencephaly | **Transport of cytoplasmic dynein** (anterograde) | Energy impairment | | Yamade, 2008 | |
|  | *DYNC1H1* (dynein) | Lysencephaly | See above | See above | | Braunstein, 2010 | |
|  | *TUBA1A*  (tubulin) | Lysencephaly | **Cytoskeleton structure, neuronal guidance, migration** |  | | Bahy-Buisson, 2008 | |
|  | *TUBA8*  Ctubulin) | Polymicrogyria with optic nerve hypoplasia, corpus callosum abnormalites | **Cytoskeleton structure, neuronal guidance, migration** |  | | Abdollahi, 2009 | |
|  | TUBB2B  Ctubulin) | Asymetrical polymicrogyria | **Cytoskeleton structure, neuronal guidance, migration** |  | | Breuss, 2012 | |
|  | **IEM with cortical migration defects, and general comments** | -**Complex molecule defects:** O-glycosylation, *PIK3R2* (phosphoinositide 3 kinase), peroxisomal defects, congenital form of CLN (cathepsin D)  **-Energy defects:** fumarase deficiency  -**Small molecule disease:** MFSD2A transport defect, asparagine synthetase deficiency  Neurogenetic cortical migration defects are related to cytoskeleton dysfunction (microtubule-related diseases). Few IEM diseases produce these changes but probably the pathophysiology interferes with these antenatal neuronal guidance processes | | | | | |
| ***Epileptic Encephalopathy***  Clinical signs:  Early age seizures, EEG paroxysmal activity, seizures that are usually multi-form and intractable, cognitive, behavioral, and neurological deficits are associated | *TRAK1* | Early onset myoclonic epilepsy and white matter abnormalities | **Mitochondrial transport** | Energy impairment, neurotransmission | | Barel, 2017 | |
|  | *KIF5A* | Progressive neonatal onset leukoencephalopathy with myoclonic seizures | **Axonal transport** | Energy impairment, neurotransmission | | Rydzanicz M, 2017 | |
|  | SV related disorders | Epilepsy and other synaptopathy symptoms | **Synaptic vesicle trafficking, interactions of lipids and proteins with the SV** | Trafficking, energy impairment, complex molecule defects (lipid remodeling) | | Cortes-Saladelafont this issue | |
|  | **IEM with Epileptic encephalopathy and general messages** | **-Small molecule diseases:** metal disorders (copper), NKH, folate defects, creatine synthesis defects, MoCo defects, serine deficiency, AADC, DPHR, SSADH, purine defects  **-Energy defects:** biotinidase, holocarboxylase synthetase, POLG, GLUT-1, MERRF  **-Complex molecule defects:** CLN, GM1, Sialidosis, peroxisomal defects, GPI anchor defects  With the exception of synaptic vesicle cycle disorders, IEM are not a major cause of epileptic encephalopathy. However, because some of them are treatable, they should be considered in the first place. Genes leading to interneuron (GABAergic) dysfunction, unbalance between glutamate and GABAergic transmission, channelopathies and some cytoskeleton disorders are amongst the most common causes of early epileptic encephalopathies | | | | | |
| ***ID+/-EPILEPSY +/- NEURO-***  ***BEHAVIORAL SIGNS*** | SV related disorders  Other synaptic dysfunctions | Synaptopathy spectrum | **Synaptic vesicle trafficking, interactions of lipids and proteins with the SV** | Trafficking, energy impairment, complex molecule defects (lipid remodeling) | | Cortes-Saladelafont , Tristán-Noguero, in this issue | |
|  | **IEM with ID + synaptopathy spectrum**  **And general messages** | **-Small molecule defects:** SSADH, creatine transport defects, purine disorders, homocystinurias, other aminoacidopathies  **-Early stages of progressive disorders** (complex molecule and energy defects)  This spectrum of symptoms is very common (probably the most typical) in any kind of genetic disorder that impairs neurodevelopment. Synaptic function is very often involved as well as dendritic arborisation. Cytoskeleton abnormalities are also common in spite of a normal macroscopic brain structure. | | | | | |
| ***Complex syndromes:***  associate diverse combinations of neurological and non-neurological signs | *Myosin 5A* | Griscelli syndrome: neuroectodermal melanosomal disease | **Vesicle transport** (short distance transport)  **Trafficking** | Energy impairment, complex molecule def | | Menasche et al, 2005 | |
|  | *AP3B1,BLOC153,DTNBP1* | Hermansky-Pudlak syndrome types 2,8,7 | **Trafficking**  coat adapter vesicle defect | Lysome biogenesis | | De Matteis, 2011 | |
|  | *OCRL* | Lowe, Dent2 | **Trafficking** | Phosphoinositide phosphatase deficiency | | Attree et al, 1999 | |
|  | *COG 1,7,8*  Glycosylation type IIg, e h | Costovertebral abnormalities, dysmorphic features, cerebellar atrophy | **Trafficking**  **(tethering defects)** | Glycosylation defects | | De Matteis, 2011 | |
|  | *EPG5* | Vici syndrome | **Autophagy impairment**  Autophagosome-lysosome fusion | Complex molecule def. | | Maday, 2014 | |
|  | *WDR45* | BPAN: NBIA subtype, Rett-like features | **Autophagy impairment**  Autophagosome formation | Complex molecule def. | | Haack et al, 2012 | |
|  | *SNAP29* | CEDNIK neurocutaneous syndrome | **Trafficking**  Intracellular vesiculation | Complex molecule def. | | Sprecher et al, 2005 | |
|  | **IEM complex syndromes** | In general related to **complex molecule defects.**  **Most of these complex syndromes belong to new categories of IEMs involving intracellular vesiculation, trafficking, processing of complex molecules, and quality control processes (like protein folding and autophagy).** Other than CEDNIK and Vici syndromes (in the table), mutations in *AP5Z1 (mimicking* LSDs),  Rabenosyn-5 (defective endocytic trafficking ) and other synaptic vesicle related mutations, belong also to this category of diseases. | | | | | |
| *Neurosenso-rial defects* | **General messages** | Hypoacusia and optic atrophy are common signs in neurodegenerative disorders involving axonal dysfunction. There are several genes related to deafness and mutations in the genes Myosin I, III, VI, XIV, XV, involved in trafficking and cytoskeleton abnormalities.  Other ocular abnormalities such as cataracts and retinitis pigmentosa are more common in IEM (in particular, in those related to energy defects and complex molecule defects: complex lipid synthesis and remodelling diseases, lysosomal disorders). Optic nerve atrophy is found in energy defects (mitochondrial diseases, PDH, biotinidase deficiency), homocysteine remethylation defects (intoxication) and different causes of metabolic leukodystrophies | | | | | |

**Table 2**. **Major neurological syndromes and their corresponding cell biology mechanisms and metabolic impairment (cellular neurometabolism approach)**

This table contains diseases that are representative of well-defined cell biology mechanisms which can be “easily” linked to pathophysiological categories described in IEM. This is not an exhaustive list of genes for every category of major neurological syndromes and does not describe in detail the clinical features of every disease.

**Abbreviations**

AA: amino acids; ADL: adrenoleukodystrophy; ALS : Amiotrophic lateral sclerosis (progressive muscle weakness and paralysis by motor neuron degeneration); AMN: adrenomyeloneuropathy; AR: autosomal recessive. BDNF: brain derived neurotrophic factor; BPAN: Beta-propeller protein associated neurodegeneration. CDG: congenital disorders of glycosylation; CEDNIK: cerebral dysgenesis, neuropathy, ichthyosis, and palmoplantar keratoderma syndrome; CLN: Ceroid lipofuscinosis; CHMP2B: charged multivesicular body protein 2B; CMT: Charcot-Marie-Tooth; DA: dopamine; Def.: defects; CPT2: Carnitine palmitoyl tyransferase 2; CTX: Cerebrotendinous xanthomatosis; ERS: Endoplasmic Reticulum Stress; ETFDH: Electron transfer flavoprotein deshydrogenase;FAD: Flavin adenine dinucleotide; FTD: frontotemporal dementia; HHH: Hyperammonaemia, hyperornithinaemia, homocitrullinaemia syndrome ; HSP: hereditary spastic paraparesis, ID: intellectual disability; LCHAD: Long chain hydroxyl acylCoA deshydrogenase; MNGIE :Mitochondrial neurogastrointestinal encephalopathy; NBIA: Neurodegenration with brain iron accumulation; OXPHOS Oxidative phosphorylation ;PDH: Pyruvate deshydrogenase; PMA: progressive muscle atrophy due to motor neuron degeneration; SBMA: spinal and bulbar muscle atrophy due to motor neuron degeneration; SMA: spinal muscle atrophy; TARDBP: TAR DNA-binding protein-43; SCA: spinocerebellar ataxia; Perry syndrome: Parkinsonism, psychiatric changes, weight loss, and abnormally slow breathing (hypoventilation). SV: synaptic vesicle. TF: trifunctional enzyme; UPR: unfolded protein response. VAPB: vesicle associated membrane protein

**REFERENCES TABLE S1**

Abbas, N., Lucking, C. B., Ricard, S. et al (1999) . A wide variety of mutations in the parkin gene are responsible for autosomal recessive parkinsonism in Europe. Hum. Molec. Genet. 8: 567-574.

Abdollahi, M. R., Morrison, E., Sirey, T. et al (2009) Mutation of the variant alpha-tubulin TUBA8 results in polymicrogyria with optic nerve hypoplasia. Am. J. Hum. Genet. 85: 737-744

Akizu, N., Cantagrel, V., Zaki, M. S et al (2015) Biallelic mutations in SNX14  cause a syndromic form of cerebellar atrophy and lysosome-autophagosome dysfunction. Nature Genet. 47: 528-534, 2015.

Aliaga, L., Lai, C., Yu, J. et al (2013) Amyotrophic lateral sclerosis-related VAPB P56S mutation differentially affects the function and survival of corticospinal and spinal motor neurons. Hum. Molec. Genet. 22: 4293-4305

Attree, O., Olivos, I. M., Okabe, I. et al (1992) The Lowe's oculocerebrorenal syndrome gene encodes a protein highly homologous to inositol polyphosphate-5-phosphatase. Nature 358: 239-242

Bahy-Buisson, N., Poirier, K., Boddaert, N., et al (2008) Refinement of cortical dysgeneses spectrum associated with TUBA1A mutations. J. Med. Genet. 45: 647-653

Bansal, D., Miyake, K., Vogel, S. et al (2003). Defective membrane repair in dysferlin-deficient muscular dystrophy.Nature 423: 168-172

Barel O, Christine V Malicdan M, Ben-Zeev B, et al (2017). Deleterious variants in TRAK1 disrupt mitochondrial movement and cause fatal encephalopathy”. Brain, 140 (3): 568–581

Baxter, R. V., Ben Othmane, K., Rochelle, J. M., et al. (2002). Ganglioside-induced differentiation-associated protein-1 is mutant in Charcot-Marie-Tooth disease type 4A/8q21. Nature Genet. 30: 21-22

Benajiba, L., Le Ber, I., Camuzat, A., et al (2009). French Clinical and Genetic Research Network on Frontotemporal Lobar Degeneration/Frontotemporal Lobar Degeneration with Motoneuron Disease. TARDBP mutations in motoneuron disease with frontotemporal lobar degeneration. Ann. Neurol. 65: 470-474

Bernard G., Chouery E., Putorti M. L. et al (2012) Mutations of POLR3A encoding a catalytic subunit of RNA polymerase pol III cause a recessive hypomyelinating leukodystrophy. Am. J. Hum. Genet. 89: 415-423, 2011. Note: Erratum: Am. J. Hum. Genet. 91: 972

Bitoun, M., Bevilacqua, J. A., Prudhon, B., et al (2007) Dynamin 2 mutations cause sporadic centronuclear myopathy with neonatal onset. Ann. Neurol. 62: 666-670

Blondeau, F., Laporte, J., Bodin, S., et al (2000). Superti-Furga, G., Payrastre, B., Mandel, J.-L.Myotubularin, a phosphatase deficient in myotubular myopathy, acts on phosphatidylinositol 3-kinase and phosphatidylinositol 3-phosphate pathway. Hum. Molec. Genet. 9: 2223-2229

Bogershausen, N., Shahrzad, N., Chong, J. X. et al (2013) Recessive TRAPPC11mutations cause a disease spectrum of limb girdle muscular dystrophy and myopathy with movement disorder and intellectual disability. Am. J. Hum. Genet. 93: 181-190

[Bonifati V](https://www.ncbi.nlm.nih.gov/pubmed/?term=Bonifati%20V%5BAuthor%5D&cauthor=true&cauthor_uid=14598065)1, [Rizzu P](https://www.ncbi.nlm.nih.gov/pubmed/?term=Rizzu%20P%5BAuthor%5D&cauthor=true&cauthor_uid=14598065), [Squitieri F](https://www.ncbi.nlm.nih.gov/pubmed/?term=Squitieri%20F%5BAuthor%5D&cauthor=true&cauthor_uid=14598065), et al (2003). DJ-1(PARK7), a novel gene for autosomal recessive, early onset parkinsonism. Neurol Sci.;24(3):159-60.

Braunstein, K. E., Eschbach, J., Rona-Voros, K., et al (2010) A point mutation in the dynein heavy chain gene leads to striatal atrophy and compromises neurite outgrowth of striatal neurons. Hum. Molec. Genet. 19: 4385-4398

Breuss, M., Heng, J. I.-T., Poirier, K., et al. (2012) Mutations in the beta-tubulin gene TUBB5 cause microcephaly with structural brain abnormalities. Cell Rep. 2: 1554-1562

Bruggemann, N., Hagenah, J., Reetz, K. et al (2010) , Recessively inherited parkinsonism: effect of ATP13A2 mutations on the clinical and neuroimaging phenotype. Arch. Neurol. 67: 1357-1363

Butler, R., Leigh, P. N., McPhaul, M. J et al. (1998) Truncated forms of the androgen receptorare associated with polyglutamine expansion in X-linked spinal and bulbar muscular atrophy. Hum. Molec. Genet. 7: 121-127, 1998

Calvo, J., Funalot, B., Ouvrier, R. A., et al (2009) Genotype-phenotype correlations in Charcot-Marie-Tooth disease type 2 caused by mitofusin 2 mutations. Arch. Neurol. 66: 1511-1516

Chen, Y., Dorn, G. W., II. (2013) PINK1-phosphorylated mitofusin 2 is a parkin receptor for culling damaged mitochondria. Science 340: 471-475,

Chow, C. Y., Landers, J. E., Bergren, S. K.,et al (2009). Deleterious variants of  FIG4, a phosphoinositide phosphatase, in patients with ALS.Am. J. Hum. Genet. 84: 85-88

Claeys, K. G., Zuchner, S., Kennerson, M., et al (2009). Phenotypic spectrum of dynamin 2 mutations in Charcot-Marie-Tooth neuropathy. Brain 132: 1741-1752

Crimella, C., Baschirotto, C., Arnoldi, A.,et al (2012)  Mutations in the motor and stalk domains of KIF5A in spastic paraplegia type 10 and in axonal Charcot-Marie-Tooth type 2. Clin. Genet. 82: 157-164

De Matteis MA, Luini A. (2011). [Mendelian disorders of membrane trafficking.](https://www.ncbi.nlm.nih.gov/pubmed/21899453) N Engl J MedSep 8;365(10):927-38

Deng, H.-X., Hentati, A., Tainer, J. A., et al (1993) Amyotrophic lateral sclerosis and structural defects in Cu,Zn superoxide dismutase. Science 261: 1047-1051

Devon RS, Orban PC, Gerrow K, et al (2006). Als2-deficient mice exhibit disturbances in endosome trafficking associated with motor behavioral abnormalities. Proc Natl Acad Sci U S A. 20;103(25):9595-600.

Drew, A. P., Zhu, D., Kidambi, A., Ly, C.,et al (2015). Improved inherited peripheral neuropathy genetic diagnosis by whole-exome sequencing. Molec. Genet. Genomic Med. 3: 143-154

Eschbach, J., Sinniger, J., Bouitbir, J., et al. (2013). Dynein mutations associated with hereditary motor neuropathies impair mitochondrial morphology and function with age. Neurobiol. Dis. 58: 220-230

Farrer, M. J., Hulihan, M. M., Kachergus, J. M., et al (2009)..DCTN1 mutations in  Perry syndrome.  Nature Genet. 41: 163-165, 2009.

Galbiati, F., Volonte, D., Minetti, C., et al (1999).  Phenotypic behavior of caveolin-3 mutations that cause autosomal dominant limb girdle muscular dystrophy (LGMD-1C): retention of LGMD-1C caveolin-3 mutants within the Golgi complex. J. Biol. Chem. 274: 25632-25641

Gauthier, L. R., Charrin, B. C., Borrell-Pages, M., et al (2004). Huntingtin controls neurotrophic support and survival of neurons by enhancing BDNF vesicular transport along microtubules. Cell 118: 127-138

Haack, T. B., Hogarth, P., Kruer, M. C., et al (2012). Exome sequencing reveals de novo WDR45 mutations causing a phenotypically distinct, X-linked dominant form of NBIA. Am. J. Hum. Genet. 91: 1144-1149

Kim, M., Sandford, E., Gatica, D., et al.(2016)  Mutation in ATG5 reduces autophagy and leads to ataxia with developmental delay. eLife 5: e12245

Lalani, S. R., Liu, P., Rosenfeld, J. A., et al (2016). Recurrent muscle weakness with rhabdomyolysis, metabolic crises, and cardiac arrhythmia due to bi-allelic TANGO2mutations. Am. J. Hum. Genet. 98: 347-357

Levy JR, Sumner CJ, Caviston JP, et al. (2006). A motor neuron disease-associated mutation in p150Glued perturbs dynactin function and induces protein aggregation. J Cell Biol;172:733–745.

Lyons DA1, Naylor SG, Scholze A, et al (2009). Kif1b is essential for mRNA localization in oligodendrocytes and development of myelinated axons. Nat Genet. 41(7):854-8. doi: 10.1038/ng.376. Epub 2009 Jun 7.

Maday S, Holzbaur EL. (2014). Autophagosome biogenesis in primary neurons follows an ordered and spatially regulated pathway. Dev Cell 30(01):71–85

Marszalek, J. R., Weiner, J. A., Farlow, S. J., et al (1999). Novel dendritic kinesin sorting identified by different process targeting of two related kinesins: KIF21A and KIF21B.J. Cell Biol. 145: 469-479

Menasche, G., Ho, C. H., Sanal, O., et al (2005). Griscelli syndrome restricted to hypopigmentation results from a melanophilin defect (GS3) or a MYO5A F-exon deletion (GS1). J. Clin. Invest. 112: 450-456, 2003. Note: Erratum: J. Clin. Invest. 115: 1100

Muller, B., Melki, J., Burlet, P.,et al (1992). Proximal spinal muscular atrophySMA types II and III in the same sibship are not caused by different alleles at the SMA locus on 5q. Am. J. Hum. Genet. 50: 892-895

Munch, C., Sedlmeier, R., Meyer, T., et al (2004). Point mutations of the p150 subunit of dynactin (DCTN1)gene in ALS. Neurology 63: 724-726

Miyatake, S., Osaka, H., Shiina, M., et al (2014). Expanding the phenotypic spectrum of  TUBB4A-associated hypomyelinating leukoencephalopathies. Neurology 82: 2230-2237

Numata, Y., Morimura, T., Nakamura, S., et al (2013). Depletion of molecular chaperones from the endoplasmic reticulum and fragmentation of the Golgi apparatus associated with pathogenesis in Pelizaeus-Merzbacher disease. J. Biol. Chem. 288: 7451-7466,

Orlacchio, A., Babalini, C., Borreca, A.,et al (2010). SPATACSIN mutations cause autosomal recessive juvenile amyotrophic lateral sclerosis. Brain 133: 591-598, 2010

Pearson, J. P., Williams, N. M., Majounie, E., et al (2011). Familial frontotemporal dementia with amyotrophic lateral sclerosis and a shared haplotype on chromosome 9p. J. Neurol. 258: 647-655

Poirier K, Saillour Y, Bahi-Buisson N, et al (2010). Mutations in the neuronal ß-tubulin subunit TUBB3 result in malformation of cortical development and neuronal migration defects. Hum Mol Genet. 15;19(22):4462-73.

Rydzanicz M, Jagła M, Kosinska J, et al (2017) “KIF5A de novo mutation associated with myoclonic seizures and neonatal onset progressive leukoencephalopathy” Clinical Genetics, 91(5):769-773.

Smith, B. N., Ticozzi, N., Fallini, C., et al (2014). Exome-wide rare variant analysis identifies TUBA4A  mutations associated with familial ALS. Neuron 84: 324-331

Sprecher, E., Ishida-Yamamoto, A., Mizrahi-Koren, M.,et al (2005).  A mutation in SNAP29, coding for a SNARE protein involved in intracellular trafficking, causes a novel neurocutaneous syndrome characterized by cerebral dysgenesis, neuropathy, ichthyosis, and palmoplantar keratoderma. Am. J. Hum. Genet. 77: 242-251,

Tischfield, M. A., Baris, H. N., Wu, C., et al (2010). Human TUBBE 3 mutations perturb microtubule dynamics, kinesin interactions, and axon guidance. Cell 140: 74-87

Tosch, V., Rohde, H. M., Tronchere, H et al (2006). A novel PtdIns3P and PtdIns(3,5)P2 phosphatase with an inactivating variant in centronuclear myopathy. Hum. Molec. Genet. 15: 3098-3106

Urwin, H., Authier, A., Nielsen, J. E.,et al (2010). Disruption of endocytic trafficking in frontotemporal dementia with CHMP2B mutations. Hum. Molec. Genet. 19: 2228-2238

Yamada K, Andrews C, Chan WM, et al (2003). KIF21A in congenital fibrosis of the extraocular muscles type 1 (CFEOM1) Nature Genetics volume 35, pages 318–321

Yamada M1, Toba S, Yoshida Y, et al (2008). LIS1 and NDEL1 coordinate the plus-end-directed transport of cytoplasmic dynein. EMBO J. 8;27(19):2471-83.

Yan, J., Deng, H.-X., Siddique, N.,et al (2010). Frameshift and novel mutations in FUS in familial amyotrophic lateral sclerosis and ALS/dementia. Neurology 75: 807-814, 2010.

Zhao, C., Takita, J., Tanaka, Y. ET AL (2001) Charcot-Marie-Tooth disease type 2A caused by mutation in a microtubule motor KIF1B-beta. Cell 105: 587-597
