# Supplementary figures and images for: Cellular neurometabolism: a tentative to connect cell biology and metabolism in neurology
Source: J Inherit Metab Dis. 2018 Jul 16;41(6):1043–54. doi: 10.1007/s10545-018-0226-8 (PMC6326994; doi:10.1007/s10545-018-0226-8)

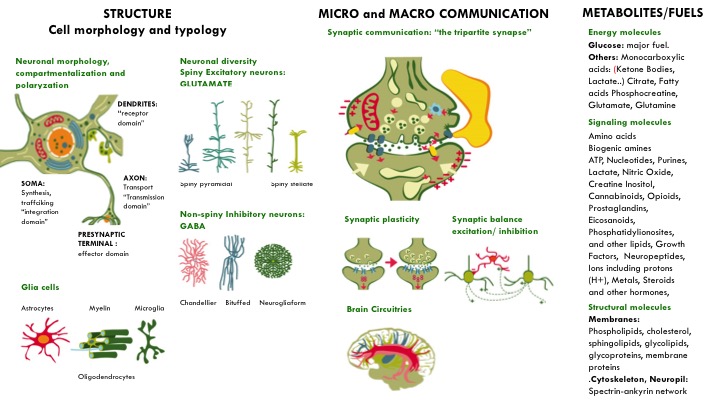

Supplement: Supplementary file 2 — Main cell types, biological functions and metabolites in the brain. Cell morphology and typology: the neocortex comprises two major cell types: neurons and glia. Neurons are the signalling cells of the nervous system, and glia perform myriad functions to support the function of neurons. Neurons can be further subdivided into excitatory projection neurons (glutamatergic)and inhibitory interneurons (Gabaergics). Only some examples of GABAergic cells are provided. Micro and Macro-communication refers to networks of brain cell communication at the synaptic level and in the organisation or brain circuitries. Further explanations about the main synaptic biological functions and the role of metabolites in the brain are provided throughout the text. (JPG 90 kb) [file 10545_2018_226_MOESM2_ESM.jpg]

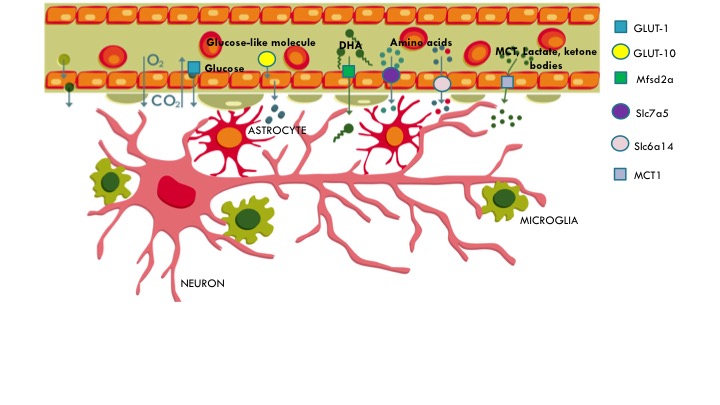

Supplement: Supplementary file 3 — Blood-brain-barrier (composed by endothelial cells and astrocytes) and transporters related to IEM. NEUROMETABOLIC DISEASES LINKED TO BLOOD BRAIN BARRIER (BBB) DEFECTS: Of the 14 GLUTs transporters, only GLUT1 and GLUT-10 are related with brain diseases. Lactate: - taken up from circulation via MCT1(expressed in endothelial cells, astrocytes and oligodendrocytes); −produced by glycolytic astrocytes and oligodendrocytes; −secreted via MCT1 and MCT4. The neuron-specific MCT2 mediates lactate uptake into the neurons. Of the 22 amino acid transporters at the endothelial cells in the BBB, only 2 are related with diseases. BCAA (branched chain amino acid) defect mimicks BCDHK (branched chain dehydrogenase kinase) defect; SLC6a14 transporter is expressed in astrocytes and release serine trom the astrocytic synthesis of serine and is also expressed at the endothelial cells of the BBB although with poor permeability. Among the Lipid transporters in the BBB (lipoprotein receptors, ABC transporters and fatty acid transporters), only one recently described defect, Mfsd2a mutations, is related with human disease.). The brain does not synthesise DHA, which is imported across the BBB through the Major Facilitator Superfamily Domain 2a (Mfsd2a). Mfsd2a transports DHA (docohexanoic acid) as well as other fatty acids in the form of lysophosphatidylcholine (LPC). (JPG 67 kb) [file 10545_2018_226_MOESM3_ESM.jpg]
